# Management and outcome of true visceral and renal artery aneurysm repair

**DOI:** 10.1007/s00423-021-02149-1

**Published:** 2021-03-23

**Authors:** Steffen Wolk, Marius Distler, Christoph Radosa, Florian Ehehalt, Hendrik Bergert, Jürgen Weitz, Christian Reeps, Stefan Ludwig

**Affiliations:** 1 Department of Visceral, Thoracic and Vascular Surgery, Medizinische Fakultät Carl Gustav Carus, Technische Universität Dresden, Dresden, Germany; 2 Institute for Diagnostic and Interventional Radiology, Medizinische Fakultät Carl Gustav Carus, Technische Universität Dresden, Dresden, Germany; 3 Department of Vascular and Endovascular Surgery, HELIOS Klinikum, Erfurt, Germany

**Keywords:** Visceral artery aneurysm,Renal artery aneurysm, Endovascular therapy, Open surgical therapy

## Abstract

**Purpose:**

Visceral and renal artery aneurysms (VAA, RAA) are very rare pathologies. Both surgical and endovascular therapies are discussed as therapeutic options for ruptured and non-ruptured aneurysm repair; we describe our experience in the open and endovascular management of these entities.

**Methods:**

Retrospective database analysis of 60 treated VAA and RAA in 59 patients between 1994 and 2020. Outcome data was descriptively analyzed.

**Results:**

Thirty-seven aneurysms were surgically treated and 23 interventionally. In the total study cohort, we observed a mortality of 1.7% and a morbidity of 18.6%. One major complication occurred. The morbidity was higher after surgical repair in ruptured and non-ruptured cases. The mean aneurysm diameter was 30.5 ± 15.6 mm. Patients with hepatic or pancreaticoduodenal artery aneurysms presented more often in the stage of rupture, without differences in aneurysm size. The length of hospital stay after endovascular repair was significantly shorter compared to open surgical treatment (7.2 ± 6.9 days versus 11.8 ± 6.7 days, *p* = 0.014), but only in elective cases. Primary technical success was significantly better in patients that underwent surgical repair in an intention to treat analysis (100% versus 79.3%). The mean follow-up of the cohort was 53.5 months (range 3–207 months).

**Conclusion:**

Elective endovascular therapy and open surgery of VAA and RAA are safe procedures with a good periprocedural and long-term outcome. Surgical revascularization showed a better primary technical success but was associated with longer length of hospital stays.

## Introduction

Visceral (VAA) and renal artery aneurysms (RAA) are rare vascular pathologies. The estimated incidence is reported to be from 0.1 to 0.2% in routine autopsies for visceral and less than 1% for renal artery aneurysms [1]. They are classified by true visceral and renal artery aneurysms (all three layers of the arterial wall distended) and visceral artery pseudoaneurysms (VAPA), with bulging of the adventitial wall after a breach of the inner wall, which is important regarding pathogenesis, clinical presentation, and natural history. The formation of true VAA and RAA is mainly caused by atherosclerosis (32%) and media degeneration (24%) but also by rare diseases such as connective tissue disorders or conditions with an increased intraportal blood pressure, which should be also considered [2, 3]. Most cases are diagnosed incidentally, without any clinical symptoms, by computed tomography for other reasons [4]. Ruptured aneurysms are associated with a high mortality rate, ranging between 20 and 50%, depending on the localization [5]. Therefore, the primary aim and intention for therapy is the prevention of this devastating complication. The risk of rupture differs between its localization. The estimated lifetime risk is 3–6% in renal artery aneurysms, 2–10% in splenic artery aneurysms, 14–80% in hepatic artery aneurysms, and 75% in aneurysms of the pancreaticoduodenal artery [2, 6–8]. Furthermore, the incidence of rupture seems highly increased during pregnancy [1, 9]. The main therapeutic intention is to exclude the aneurysm (VAA/VAPA) from blood flow without impairing any end-organ perfusion. The option of choice depends mostly on morphological criteria. In previous reports, the results of endovascular and surgical repair of non-ruptured VAA/RAA were comparable, showing low mortality and morbidity under elective conditions, but mixing often different etiologies like VAPA and true aneurysms in addition to a missing long-term follow-up [10, 11].

The aim of our study was to review our results critically after open and endovascular elective and ruptured true VAA/RAA repair and to compare clinical, morphological, and therapeutic features.

## Methods

### Data collection and study population

All patients treated by surgical and endovascular means for true renal and visceral artery aneurysms between November 1994 and February 2020 were prospectively recorded in the Department of Visceral, Thoracic and Vascular Surgery at the Carl Gustav Carus University Hospital, Dresden. The data of each case was analyzed retrospectively. In accordance with the guidelines for human subject research, approval was obtained from the ethics committee at the TU Dresden (decision number EK 407102016). VAPA were excluded from the analysis due to the different etiologies and natural histories. Differentiation between VAPA and true aneurysms were made by clinical and imaging criteria. All patients received a computed tomography angiography (CT-A) scan before operation or intervention. Aneurysms with irregular arterial wall together with evidence of inflammation in the surrounding tissue or focal disruption of the vessel wall in an otherwise normal artery were classified as VAPA. Additionally, clinical criteria such as intraabdominal or retroperitoneal inflammation (e.g., pancreatitis, abscess) or malignancy, surgical or endoscopic biliary tract interventions, or previous abdominal trauma were considered.

### Indications and surgical/interventional technique

Indication for treatment of VAA/RAA was a diameter greater than 2.5 cm for true aneurysms, a growth rate greater than 0.5 cm/year, symptoms caused by the aneurysm, or rupture according the ESVS guidelines [12]. Elective cases were reviewed prior to treatment at our interdisciplinary vascular board, and a therapeutic method was chosen by morphological criteria individually for each patient. All patients received a thin-sliced 1-mm computed tomography angiography (CT-A) scan for treatment planning. The endovascular approach was favored because of less invasiveness. Exclusion criteria for endovascular treatment were tortuosity of the target vessel, the origin of important branch vessels from the aneurysm, or the insufficient sealing zone for stent graft placement. In emergency situations, a decision was made by the vascular surgeon and the interventional radiologist on-call, based on the severity of illness and urgency.

For open surgical treatment, a midline or transverse laparotomy for exposure of the VAA/RAA was performed. The pathologies were excluded by resection with end-to-end reanastomosis, interposition graft, or bypass. Great saphenous vein was preferred as bypass material. In cases where no autologous material was present, prosthetic grafts were used. In cases of rupture, organ resection (e.g., splenectomy in the case of splenic artery aneurysm) was the most often performed technique.

For endovascular treatment, a percutaneous transfemoral or transbrachial access was used depending on the desired treatment (coil embolization or stent graft) as well as the angle between the visceral arteries and aorta. After a size 4 French sheath was introduced using the Seldinger technique into the femoral or brachial artery, selective angiograms of the superior mesenteric artery, the coeliac trunk, or the renal arteries were performed. Depending on the location of the VAA, coil embolization and stent graft implantation were the preferred techniques for endovascular treatment. The choice of treatment was at the discretion of the interventional radiologist. In general, peripheral arteries or arteries with an adequate collateral flow were treated with coil embolization, whereas stent grafts were implanted in main arteries such as the hepatic and proximal mesenteric artery to preserve the vascular flow and avoid organ dysfunction. We used two different types of coils, the AZUR® CX Peripheral Coil System (Terumo Medical Corporation, Somerset, NJ) or the Tornado® Embolization Coil (Cook incorporated, Bloomington, IN), and one type of stent graft, the VIABAHN® Endoprosthesis (W. L. Gore & Associates, Inc; Flagstaff, AZ) since 2009. In case of stent graft treatment, a long size 5 to 8 French sheath (depending on the type and diameter of the stent graft) was introduced into the vessel ostium, and a 0.035′′ stiff guidewire was placed in a peripheral branch of the artery. After determination of the adequate diameter and length in the preinterventional CT and the angiography, the stent graft was placed into the vessel and released. If coil embolization was necessary, the artery was catheterized using a 0.018′′ guidewire. In arteries with collateral flow, the microcatheter was placed distal to the VAA to perform a “frontdoor-backdoor” coil embolization or a coil packing of the VAA.

### Outcome parameters and definitions

The main outcome parameter of the study was the assessment of in-hospital mortality as well as the overall morbidity. All perioperative complications were recorded. Major complications were defined as any complication needing re-operation or re-intervention. Further outcome parameters were primary technical success, the need for re-intervention after aneurysm repair during the follow-up, and the length of hospital stay. Length of hospital stay was defined as days from surgery or intervention until discharge from the hospital. Primary technical success of endovascular therapy was defined according to the reporting standards for EVAR of the Society for Vascular Surgery on an intention to treat basis. It requires the successful introduction and deployment of the device (coil, stent graft) in the absence of surgical conversion or mortality, persistent contrast filling of the aneurysm in the completion angiography, or in case of stent graft deployment, an obstruction [13].

Patients were seen in the outpatient clinic during follow-up periods of 3-month, 9-month, and yearly thereafter, whenever possible, but 15 patients were lost during follow-up. CT-A scans were performed in any such case where an ultrasound examination was not diagnostically conclusive.

### Statistical analysis

The statistical analysis was performed using SPSS for Mac, version 21.0 (SPSS, Inc., Chicago, IL). All clinical characteristics were grouped to build categorical or nominal variables. Dichotomous variables were recorded as absolute frequencies (number of cases) and relative frequencies (percentages). All outcome parameters between surgical and endovascular treated patients were analyzed as treated. The primary technical success for endovascular treatment was analyzed on an intention to treat basis. Continuous data were presented as mean and standard deviation if not indicated otherwise. Univariate examination relationships were performed with a χ^2^-test. A two-sided *p* value < 0.05 was considered statistically significant.

## Results

### Patient characteristics

Sixty true aneurysms in 59 patients were identified. Thirty patients were male (50.8%). The average age of the cohort was 62.8 ± 13.3 years. The most common comorbidity was hypertension, followed by hyperlipidemia, smoking history, coronary heart disease, diabetes, and chronic obstructive pulmonary disease (COPD) (Table 1).

**Table 1:** Patient characteristics

	*n* = 59	%/std
Male	30	50.8
Age (years)	62.8	13.3
Concomitant aortic aneurysm	7	11.9
Hypertension	46	80
Dyslipidaemia	21	35.6
Smoker	8	13.6
Soronary heart disease	7	11.9
Diabetes	5	8.5
COPD	2	3.4
Connective tissue disorder	1	1.7

Legend: *VAA* visceral artery aneurysm, *COPD* chronic obstructive pulmonary disease

### Aneurysm characteristics and clinical presentation

The mean aneurysm diameter was 30.5 ± 15.6 mm. There was no significant difference of the diameter between ruptured and non-ruptured aneurysms (30.0 ± 11.2 mm versus 31.7 ± 23.3 mm, *p* = n.s.). The aneurysms were located in the splenic artery (36.7%), the hepatic artery (15%), the renal artery (13.3%), the pancreaticoduodenal artery (8.3%), the gastroduodenal artery (8.3%), the celiac trunk (8.3%), the left gastric artery (3.3%), the superior mesenteric artery (3.3%), and the inferior mesenteric artery (3.3%) (Table 2). Patients with pancreaticoduodenal, celiac trunk, left gastric artery, and hepatic artery aneurysms had more often aneurysm rupture at time of presentation (Table 3). The mean aneurysm size in this group was 27.9 ± 15.7 mm versus 32.6 mm ± 15.5 mm (*p* = n.s.).

**Table 2:** VAA localization

	*n* = 60	%
Splenic artery	22	36.7
Hepatic artery	9	15
Renal artery	8	13.3
Pancreaticoduodenal artery	5	8.3
Gastroduodenal artery	5	8.3
Coeliac trunk	5	8.3
Left gastric artery	2	3.3
Superior mesenteric artery	2	3.3
Inferior mesenteric artery	2	3.3

**Table 3:** Rupture-dependent localization of VAA

		Rupture			
		No (*n*=42)		Yes (*n*=18)	
	*n* (total number)	*n*	%	*n*	%
Splenic artery	21	19	86.4	3	13.6
Hepatic artery	9	5	55.6	4	44.4
Renal artery	8	6	75	2	25
Pancreaticoduodenal artery	5	2	40	3	60
Gastroduodenal artery	5	4	80	1	20
Coeliac trunk	5	3	60	2	40
Left gastric artery	2	0	0	2	100
Superior mesenteric artery	2	2	100	0	0
Inferior mesenteric artery	2	1	50	1	50

In 18 patients (30.5 %), a rupture of a VAA was diagnosed. All patients that were asymptomatic did not show signs of rupture, except 1 patient with a chronic, contained ruptured aneurysm, at the time of presentation (*n* = 32, 59.3%), and 24 patients showed symptoms (40.7%). Most common aneurysm-related symptoms were epigastric pain (*n* = 12, 20.3%), hemorrhage with shock (*n* = 7, 11.9%), hemobilia (*n* =3, 5.1%), hemorrhage with hemodynamical stability (*n* = 3, 5.1%), and upper GI bleeding (*n* = 1, 1.7%) (Table 4).

**Table 4:** Symptoms

	*n* = 59	%
Asymptomatic	35	59.3
Epigastric pain	12	20.3
Hemorrhage with shock	7	11.9
Hemobilia	3	5.1
Hemorrhage—stable	3	5.1
Upper GI hemorrhage	1	1.7

### Management, periprocedural outcome and outcome

**Fig. 1:**
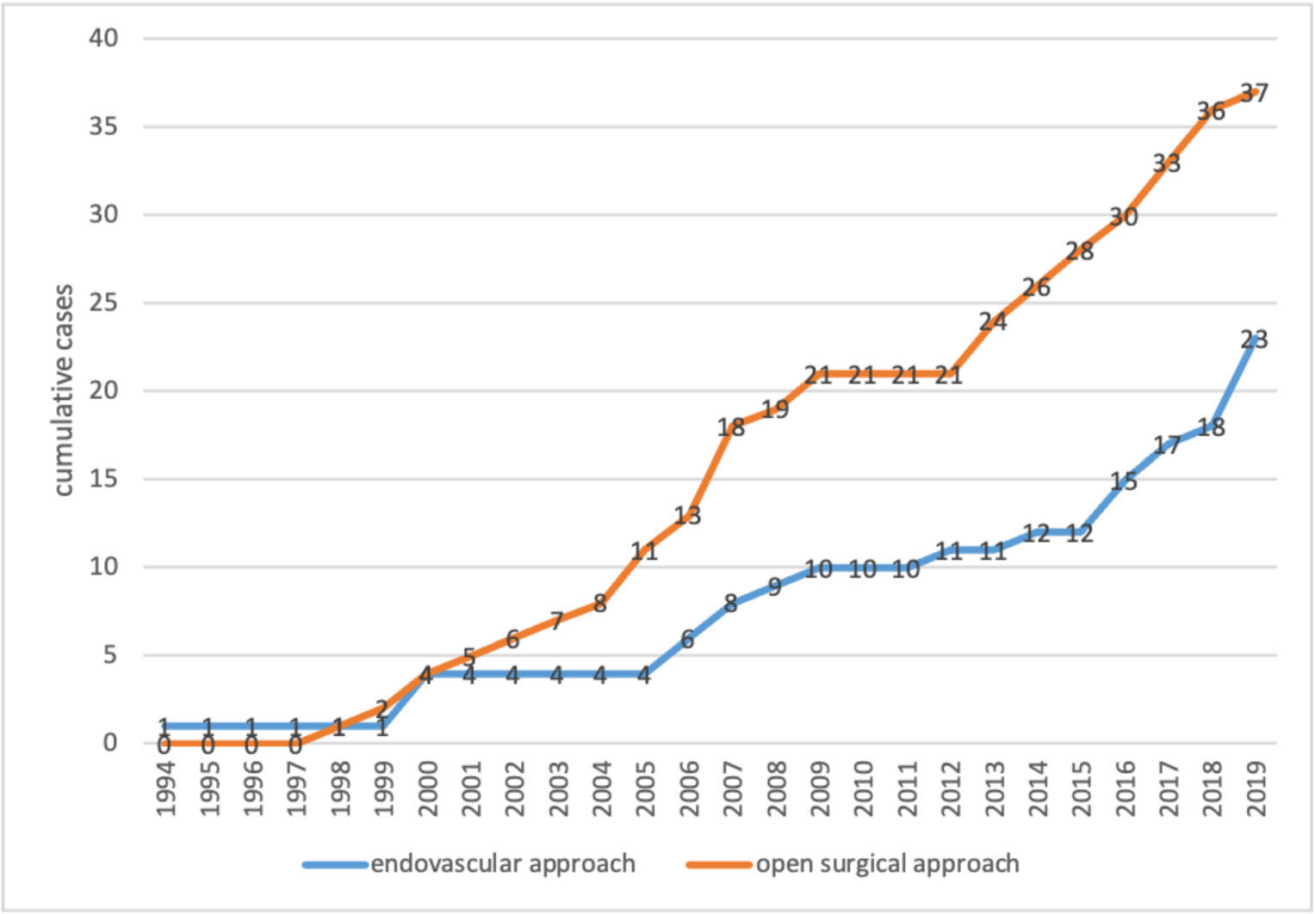
Cumulative cases of endovascular and open approach over the years 1994 to 2019

**Fig. 2:**
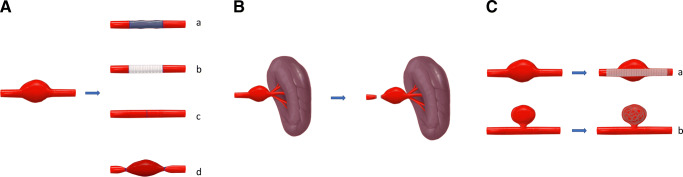
Surgical and endovascular techniques for aneurysm repair. **a** Surgical repair: (a) vein graft, (b) prosthetic graft, (c) resection with end-to-end anastomosis, (d) aneurysm ligation without revascularization; **b** end-organ resection (e.g., splenectomy); **c** endovascular repair: (a) stent graft placement, (b) coil packing. © 2020 by Cindy Fuchs. All rights reserved

Most of the aneurysms were treated surgically (open surgical treatment *n* = 37, 61.7% versus endovascular intervention *n* = 23, 38.3%). Six aneurysms in 5 patients had an unsuccessful endovascular treatment. Anatomic reasons for treatment selection are shown in Table 5. The most performed procedure in endovascular treated patients was coil embolization in 20 aneurysms (87%). Three aneurysms were repaired by stent graft placement (13%). Endovascular treatment was favored in ruptured VAA (61.1% endovascular versus 38.9% open surgical repair, *p* = 0.018). The most common open surgical procedures were resection with end-to-end anastomosis in 12 (20%) followed by vein graft interposition in 8 (13.3%), prosthetic graft interposition in 6 (10%), organ resection in 8 (13.3%), and ligation without revascularization in 3 cases (5%), respectively (Figs. 1, 2).

**Table 5:** Indications for treatment modality

		*n* = 60	%
Endovascular (*n* = 23)	Coiling: adequate collateralization of outflow	11	47.8
	Coiling: peripheral branch without risk of organ infarction	7	30.4
	Coiling: packing of saccular aneurysm without sacrifice of the outflow	2	8.7
	Stent graft	3	13
Surgical (*n* = 37)	Stent graft not available	4	10.8
	no distal sealing zone because of branch vessels	13	35.1
	No proximal sealing zone	8	21.6
	Repair together with surgery for other reasons	3	8.1
	Failed intervention	6	16.2
	Proximal vessel occlusion before aneurysm	2	5.4
	Hemodynamic instability with need for quick bleeding control	1	2.7

**Fig. 3:**
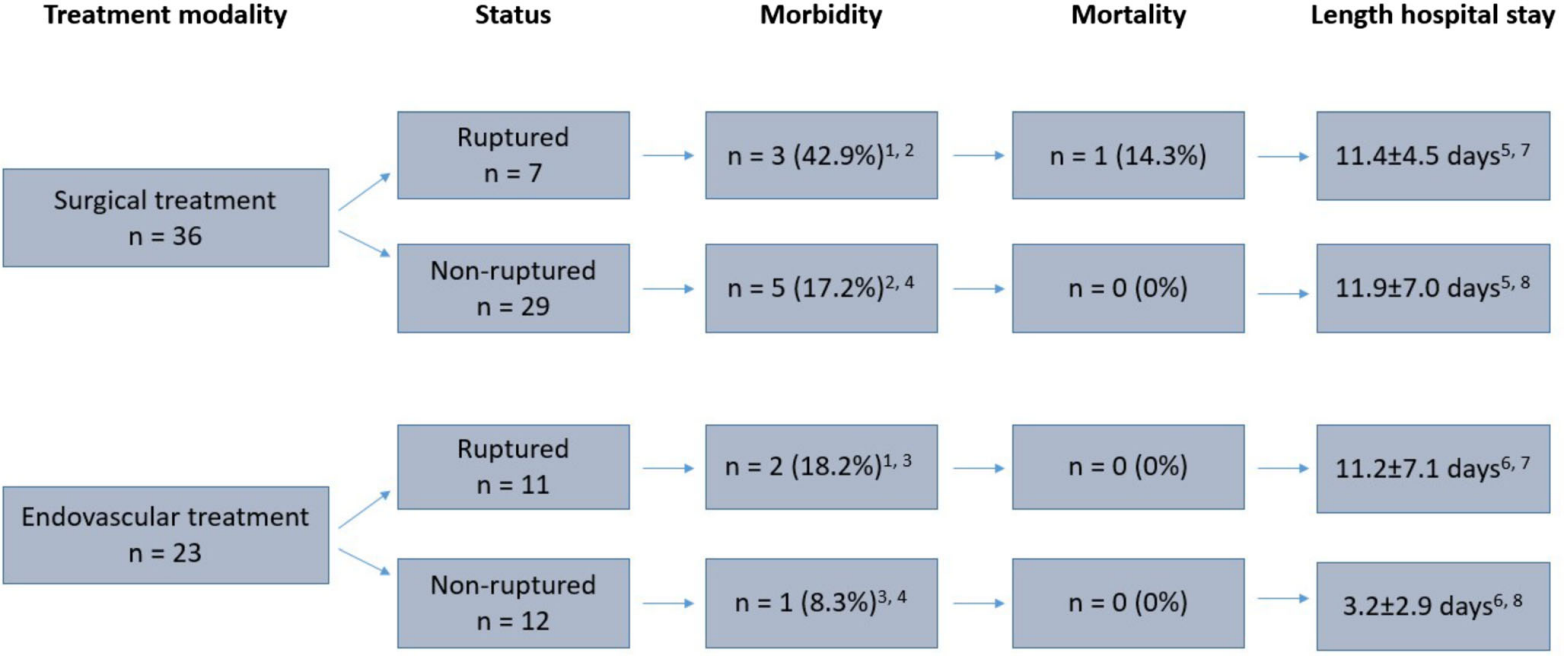
Outcome after surgical and endovascular repair in ruptured and non-ruptured cases. Thirty-seven aneurysms in 36 patients were repaired by open surgery and 23 aneurysms in 23 patients with an endovascular approach. Statistical analysis: 1: *p* = 0.25; 2: *p* = 0.14; 3: *p* = 0.48; 4: *p* = 0.46; 5: *p* = 0.83; 6: *p* < 0.01; 7: *p* = 0.93; 8: *p* < 0.01

In our cohort, we observed 1 in-hospital mortality (1.7%) in a patient after open surgical treatment of a ruptured pancreaticoduodenal artery aneurysm due to an intracranial hemorrhage in the postoperative course. The morbidity was 18.6%. None of the complications needed surgical or endovascular reintervention except one patient with myocardial infarction with need for coronary bypass surgery. The formation of a pancreatic fistula was the most common morbidity in surgically treated patients (*n* = 4). Further complications were pancreatitis (*n* = 2), postoperative delirium (*n* = 1), urinary tract infection (*n* = 1), splenic infarction (*n* = 1), and the formation of an arteriovenous fistula (*n* = 1). The morbidity after open surgical repair and after aneurysm rupture showed a tendency to be elevated but did not reached statistically significance (Fig. 3).

The average length of hospital stay in the total study cohort was 10.7 ± 7.1 days and significantly shorter after endovascular treatment (7.2 ± 6.9 days versus 11.8 ± 6.7 days, *p* = 0.014). Subgroup analysis showed that length of hospital stay was only reduced after elective endovascular compared to open repair (3.2 ± 2.9 days versus 11.9 ± 7.0 days, *p* < 0.001) (Fig. 3).

Primary technical success of the endovascular therapy (*n* = 29) on an intention to treat basis was 79.3%. Conversion to surgical therapy was indicated in 5 patients with 6 aneurysms. The reasons for these conversions were the impossibility of probing the target vessel (*n* = 2), too large of a target vessel diameter for safe stent graft deployment without risk for endoleak (*n* = 2), insufficient distal sealing zone for stent graft deployment because of branch vessels (*n* = 1), and persisting contrast filling of the aneurysm after coiling and coil dislocation (*n* = 1).

In contrast, initially surgical treated patients needed no reintervention.

During the mean follow-up of 53.5 month (range 3–207 months), 4 patients died (6.8%), but no aneurysm-related death was observed.

## Discussion

Outcome data for the repair of VAA/RAA are rare due to the low incidence. There was no gender difference in the incidence of VAA/RAA in our cohort, which is quite different from aortic aneurysms [14, 15] or popliteal artery aneurysms [16]. Shukla et al. also reported no gender difference in the incidence of repaired VAA, whereas men had a higher incidence of ruptured aneurysms [5].

The clinical practice guideline of the European Society of Vascular Surgery (ESVS) recommends, based on a low level of evidence, an endovascular repair for patients with VAA/RAA who are anatomically suitable, because of its lower morbidity and mortality [12]. In contrast, our study demonstrates acceptable perioperative morbidity and mortality for endovascular as well as open repair of VAA/RAA in the large cohort of patients at our institution.

In our study, we see no difference in aneurysm size between ruptured and non-ruptured VAA, and no advice regarding a threshold diameter in elective patients can be given. The ESVS guidelines recommend a diameter of more than 25 mm for elective repair, but exceptions of repair independent from size are recommended: aneurysms of the pancreaticoduodenal and gastroduodenal arcade, of the intra-parenchymatous hepatic arteries, in women of child-bearing age, and recipients of a liver transplant should be repaired irrespective of size [12]. Patients with pancreaticoduodenal, celiac trunk, left gastric artery, and hepatic artery aneurysms had more often aneurysm rupture at time of presentation without significant differences in aneurysm size. But missing follow-up examinations before aneurysm repair did not conduce to the statement that these patients were at higher risk for rupture. The SVS guideline from 2020 recommends a threshold diameter in asymptomatic, non-ruptured aneurysms of 30 mm in renal and splenic artery aneurysms, 20 mm in coeliac, hepatic, and jejunal/ileal arteries, and repair regardless of size in superior mesenteric, pancreaticoduodenal/gastroduodenal, and colic arteries [17].

A limitation of our study is a selection bias over this long period that begins at the dawn of endovascular therapy and ends during its height; however, the number of cumulative cases was similarly increasing over this time period. Stent grafts were available from 2009 in our center which can bias the treatment selection. We retrospectively reviewed all surgical cases before 2009. Four patients could have been treated with stent grafts (10.8%). Nevertheless, before choosing a treatment modality, a thorough study of the aneurysm morphology is crucial to prevent insufficient aneurysm exclusion or the sacrifice of viable tissue due to vessel branch coverage. This leads to the selection of open surgery for branch preservation in 56.8% of the aneurysms in this group. The study by Klausner et al. demonstrated that nearly half of RAA were located at a crucial vascular bifurcation of the renal artery, and endovascular therapy was not possible without impairing renal perfusion [18]. The advantage of endovascular therapy increases in patients with inflammatory pseudoaneurysms, hostile abdomen, and in cases of rupture [19]. In rVAA/rRAA, the distal perfusion can be sacrificed for bleeding control as a bailout manoeuver, whereas an elective situation enforces the preservation of distal perfusion. Large studies regarding covered stent placement are lacking, and the long-term patency is unclear. In a large cohort study by Pitton et al. of 253 VAA/RAA, only 23.3% (*n* = 59) of the patients received an aneurysm-specific treatment by an endovascular technique (*n* = 45) or open surgery (*n* = 14). Five patients were treated with covered stents in this series [10]. In other series, endovascular therapy by coiling was the method of choice [20]. Our data demonstrates that reconstruction with bypasses or aneurysm resection with reconstruction by end-to-end anastomosis is associated with good technical success and freedom from re-intervention. In our experience, the open surgical preservation of distal perfusion by reconstruction should be considered for elective aneurysm repair in patients who are fit for surgery.

Shukla et al. reported a technical success for endovascular therapy in 98% of the patients. In our study, the technical success of endovascular therapy in the intention to treat analysis was 79.3%. This was mostly related to the stent graft or coil not deploying due to anatomical reasons in 3 of 6 aneurysms or that the target vessel could not be probed in another 2 of 6 cases. Endovascular treatment was favored in patients with ruptured VAA/RAA. In the study by Shukla et al., a marked benefit of endovascular versus open therapy was observed in ruptured VAA/RAA. In these patients, the 30-day mortality was 7.4% versus 28.6%, respectively, but the survival of endovascular-treated patients also significantly increased compared to open repair after 2 years of follow-up (69.4% versus 46.4%) [11]. In the largest endovascular-treated cohort of Fankhauser et al. (176 patients), there was a 30-day mortality and an aneurysm-related mortality of 6.2 and 3.2%, respectively. The mortality was observed in the group of ruptured VAA/RAA without exception [20]. In our study, ruptured aneurysms were treated preferentially by endovascular intervention. This approach offers the advantage of fast bleeding control under local anesthesia. Anatomical criteria in ruptured aneurysms are secondarily relevant.

A study by Klausner et al., analyzing renal artery aneurysm treatment, demonstrated comparable data for morbidity and mortality after endovascular or open therapy but with a significantly shorter length of hospital stay in the endovascular group [18]. We also observed the benefit of endovascular therapy with a shorter length of hospital stay, but only in elective cases. It must be considered that the length of hospital stay could be decreasing over the long study period, which can bias this result. A disadvantage of the endovascular therapy is that not every aneurysm can be treated due to morphological reasons [7]. However, open therapy offers a treatment option for almost every aneurysm configuration and is associated with a low mortality of 0.5% in several studies [21, 22]. This is supported by our data, showing a low re-intervention rate in our reconstructions during the follow-up period, whereas long-term results of endovascular therapy are unclear [7]. On the other side, open surgical repair has a not significant tendency of higher morbidity after elective or emergency repair, which justifies the primary endovascular approach independent from less technical success. A considerable limitation of our retrospective analysis is that structured follow-up during this long observational period was not ensured in all patients and 15 patients were lost during follow-up.

## Conclusions

The surgical and endovascular therapies for VAA/RAA are safe procedures with a low mortality rate. Endovascular therapy is associated with a shorter length of hospital stay but offers a lower primary technical success.

## Data Availability

Not applicable

## Abbreviations

VAA: Visceral artery aneurysm
RAA: Renal artery aneurysm
VAPA: Visceral artery pseudoaneurysm
CT-A: Computed tomography angiography
